# An Observational Cohort Study of Wharton’s Jelly Tissue Allografts for Posterior Tibial Tendon Degeneration

**DOI:** 10.3390/biomedicines13102398

**Published:** 2025-09-30

**Authors:** Babak Baravarian, Gi Kwon, John Shou, Naomi Lambert, Alexis Lee, Eva Castle, Tyler Barrett

**Affiliations:** 1 University Foot & Ankle Institute, Santa Monica, CA 90403, USA; bbaravarian@mednet.ucla.edu (B.B.); gi.kwon@footankleinstitute.com (G.K.); 2 Regenative Labs, Pensacola, FL 32501, USA

**Keywords:** posterior tibial tendon, posterior tibial tendonitis, posterior tibial tendon dysfunction, tendinopathy, Wharton’s jelly, regenerative medicine

## Abstract

**Introduction:** Posterior tibial tendon dysfunction (PTTD) is a progressive degenerative tendinopathy often unresponsive to conservative care, necessitating surgical interventions with significant postoperative risks. Wharton’s jelly (WJ) tissue allograft from the human umbilical cord, with its collagen-rich matrix homologous to tendon tissue, presents a potential alternative intervention. This study aims to report preliminary findings on the safety and efficacy of WJ allografts for the supplementation of degenerated tissue in patients with PTTD. **Material and Methods:** Twenty-six patients from the observational repository were identified with PTTD (Stages II-IV) and failed at least three months of conservative care. Patients received one or two ultrasound-guided percutaneous applications of the WJ allograft. Outcomes were tracked using the Numeric Pain Rating Scale (NPRS), the Western Ontario and McMaster University Arthritis Index (WOMAC), and the Quality-of-Life Scale (QOLS) at the initial, 30, 90, and 120-day follow-ups. **Results:** The cohort was 62% male (*n* = 16) and 38% female (*n* = 10), with a mean age predominantly in the 70–89 range. From the initial to final visit (90 days for single applications, 120 days for double applications), the single-application group (*n* = 22) showed a 48.32% improvement in NPRS and a 22.73% improvement in total WOMAC. The double-application group (*n* = 8) showed a 50% improvement in NPRS and a 27.86% improvement in total WOMAC. A statistically significant improvement in NPRS was observed in the single-application group (*p* = 0.042). No adverse events were reported. **Discussion:** This study provides preliminary evidence that WJ tissue allografts may be a safe and effective minimally invasive application for degeneration of the PTT, which is associated with improvements in pain, function, and quality of life. Key limitations include a lack of a control group and a small cohort size. **Conclusions:** The positive findings of this study warrant further research through randomized controlled trials to confirm efficacy, establish optimal dosage, and compare WJ to other conservative interventions.

## 1. Introduction

Despite its clinical relevance, enthesopathy often remains unrecognized and is frequently overshadowed by more commonly diagnosed joint conditions such as arthritis. Enthesopathy is a broad term for pathologies that include ligament and tendinous injuries [1]. Repetitive mechanical stress, inflammatory responses, and micro-tearing of the local area can lead to a loss of fibrillar structure and changes in the integrity of the collagen matrix, which contribute to the thickening of the entheses [1]. One of the most common enthesopathies is tendinopathy, which is associated with damaged and diseased tendons [2]. Tendinopathy abnormalities occur in the microstructure of the tendon, disrupting the structural integrity of the collagen matrix, leading to fragmented collagen fibers and disorganized collagen bundles [2]. One condition that exemplifies these degenerative changes is posterior tibial tendon dysfunction (PTTD), which is a progressive and debilitating tendinopathy affecting the medial ankle [3]. The prevalence of PTTD ranges between 3.3% and 10%, with a higher risk associated with comorbidities, including hypertension, diabetes, and higher body mass index (BMI) [3]. Consisting of multiple divisions, the posterior tibial tendon has its primary and uniform attachment on the navicular bone, supplemented by additional connections to the medial cuneiform, lateral cuneiform, sustentaculum tali, and the bases of the metatarsals, functioning to stabilize the medial longitudinal arch and invert the midfoot [4]. The posterior tibialis tendon functions both as a plantar flexor and an inverter of the foot, which are special movements specific to the foot [5]. Posterior tibial tendon dysfunction (PTTD) defines chronic strain as the degeneration of the tendon behind and around the inner part of the ankle, causing inflammation [6]. With tendinosis, collagen degenerates in the tendon in response to chronic overuse [7]. Common symptoms of PTTD include pain behind the medial ankle, swelling, difficulty walking or standing, arch collapse, and degenerative changes in both the tendon and adjacent tissues [6,8]. These symptoms can lead to chronic microtrauma, flattening of the foot, hindfoot valgus, degeneration at the subtalar joint, and even arthritis [6,7]. The risk factors of PTTD include conditions such as overuse, high body mass index, hypertension, diabetes, corticosteroid exposure, and excessive pronation [3,9]. PTTD’s risk factors and symptoms increase the need for noninvasive or invasive procedures to aid the body in the repair process.

The determination of whether noninvasive or invasive procedures should be recommended depends on the severity of PTTD. Severity can be classified into four stages according to the Johnson and Strom classification modified by Myerson [10,11]. Stage I is defined as mild pain along the medial aspect of the ankle with characteristics of swelling, fullness, and tenderness [10]. No deformity is present in the first stage. The first stage recommends conservative management using a walking boot, physical therapy, and orthotics for around 3–4 months [11]. Stage II characterizes PTTD with moderate pain localized along a longer segment of the tendon [10]. Stage II is the first stage to present deformity of the posterior tibial tendon, showing elongation of the tendon and pronounced swelling, fullness, and tenderness [10]. PTTD in Stage II uses immobilization and physical therapy with orthotics. However, if conservative measures fail, surgical treatment such as a medial calcaneal osteotomy with posterior tendon debridement and repair is recommended [11]. Stage III describes severe pain that is evident on the lateral foot at the sinus tarsi in addition to the medial arch [10]. Elongation and disruption of the posterior tibial tendon are also present. This stage utilizes the same conservative therapies as Stages I and II, but it predominantly warrants surgical treatment such as medial double arthrodesis or triple arthrodesis because of rearfoot arthritic changes [11]. Stage IV presents PTTD with valgus deformity of the ankle and can sometimes be associated with lateral tibiotalar arthritis [10]. Stage IV is further classified into type A, which is characterized by flexible ankle deformity, or type B, which involves a fixed deformity of the ankle [10]. Individuals diagnosed with Stage IV PTTD often require surgery because of severe degeneration of the tendons in the ankle and rearfoot [11]. These surgeries include, but are not limited to, total ankle arthroplasty with replacement, deltoid ligament reconstruction, and triple arthrodesis with Achilles tendon lengthening [11]. PTTD surgery triple arthrodesis can lead to many complications, such as early osteoarthritis in the ankle joint, hardware failure, infection, and wound dehiscence [12]. The lack of successive measures taken to prevent complications or even treat PTTD highlights the importance of future research for low-risk interventions to mitigate suffering in patients.

The existing noninvasive methods for PTTD do not resolve the underlying problem of tendon degeneration and deformity, prompting the necessity of alternative approaches that could improve patient care. The patients in this case series all had evidence of structural degeneration of the posterior tibial tendon and had failed at least three months of standard conservative care. Before considering surgical intervention, patients were offered a regenerative medicine alternative with connective tissue transplantation. Wharton’s jelly (WJ) tissue is primarily composed of several different collagen fiber types, specifically types I, II, III, and V, cytokines, growth factors, and hyaluronic acid [13,14]. The most abundant collagen type in tendons is collagen I, which represents 95% of the matrix, while types III and V represent the remaining 5% [15]. The allograft used follows the FDA guidelines for minimal manipulation, maintaining all the biochemical and biophysical properties of the tissue in the final allograft. Perinatal tissues like WJ have unique biological properties like being anti-inflammatory, anti-fibrotic, anti-microbial, and immune-privileged, making them ideal low-risk transplantable tissue because of their reduced immune reactions [16]. The collagen matrix of WJ is comparable to the collagen matrix of tendons, making WJ a homologous tissue that has sufficient composition to replace damaged tissue. Due to its structural similarity to multiple native tissues, WJ is clinically applicable in various homologous anatomical sites. A recent publication on the application of WJ to damaged tendons and ligaments in the rotator cuff displays significant patient improvements in a similar use site [17]. The study demonstrated favorable outcomes in the cohort, with patients reporting improvements in pain and function, promoting overall well-being [17]. The need for alternative interventions with evidence of long-term improvements for posterior tibial tendon insufficiency is vital to increase the efficacy and safety of patient care. This preliminary observational study aims to evaluate the initial safety, feasibility, and patient-reported outcomes of percutaneous Wharton’s jelly tissue allografts applied to structural defects in the posterior tibial tendon in a treatment-resistant cohort.

## 2. Materials and Methods

### 2.1. Study Design

This is a retrospective analysis of data collected in an observational repository, conducted in accordance with the guidelines of the Declaration of Helsinki, with approval from the Institutional Review Board of the Institute of Regenerative and Cellular Medicine (IRCM-2021-311) since 2021. Informed consent was obtained from all patients prior to application. All allografts used were the 150 mg tissue product from Regenative Labs (Pensacola, FL, USA), manufactured following the FDA’s 361 guidelines for minimally manipulated human cell and tissue products. The repository design and WJ allograft processing details have been described previously [18,19]. Observer bias was minimized by having multiple observers at multiple clinical sites across the country. This analysis included only complete data sets from patients who received a single or double application of WJ tissue allografts to the posterior tibial tendon. No exclusions were made based on gender, age, body mass index (BMI), or level of initial score; however, patients were excluded if they were lost to follow-up, the data were outside the respective time range (±15 days for 30-day follow-up, ±30 days for 90-day and 120-day follow-ups), had multi-faceted defects, or received more than two applications. The cohort was further divided into two dosage groups, with 18 patients receiving one application and 8 patients receiving two applications.

### 2.2. Intervention and Patient Care Procedures

Patients displayed Posterior Tibial Tendinosis symptoms at their initial consultation, which included micro-tearing on the arch, calf tightness, Achilles tightness, tissue damage, and tendon strain. Micro-tearing is the breakage of collagen fibers along the tendon, visible via ultrasonography as disruptions in the hyperechoic lines without being a full or partial tear. All patients, defined by the Johnson and Strom classification with modification from Myerson, had PTTD in Stage II, III, or IV with evidence of structural degeneration of the tendon from ultrasound and magnetic resonance imaging (MRI). Before considering the Wharton’s jelly application, patients underwent conservative care, receiving physical therapy, shoe modifications, orthotics, and bracing. All patients had previously failed a minimum of three months of standard conservative care before being eligible to receive a WJ allograft. Prior to Wharton’s jelly application, patients received an MRI to assess the level of tissue damage. To further confirm the site of degeneration, the patients underwent a palpation and then an ultrasound to locate the deteriorating area. The patients were then injected with two cc of local anesthesia into the affected area via a 25-gauge needle under ultrasound guidance. When the area was fully prepped, the Wharton’s jelly allograft was applied to the defects with a 25-gauge needle under ultrasound guidance. Following the procedure, the patients waited thirty minutes in the office to evaluate any reactions. No adverse reactions were reported. The aftercare included restrictions on icing and anti-inflammatories for six weeks, and the patients were placed in either a boot or a brace. After two weeks, if the patients showed progress, the bracing was removed, and they could resume regular activities. Eight patients received one additional application of the WJ allograft at the 30 or 90-day mark due to limited initial progress and the severity of initial degeneration. At each follow-up, patients filled out QOLS, NPRS, and WOMAC scales to monitor their progress.

### 2.3. Outcome Measures and Statistical Analysis

Patient outcomes were assessed using the NPRS, WOMAC (Pain, Stiffness, Physical Function subscales, and Total), and QOLS at initial, 30, 90, and 120 days. In the two application groups, data sets were considered complete with one follow-up following the final application, so five of the eight patients had completed their observational period at 90 days, and three patients were complete at 120 days. To compensate for the differences in timelines, the percentage change and overall difference in scores were calculated using the last data set submitted, regardless of which day. A Kruskal–Wallis test and Chi-Squared test were performed on the study population’s baseline characteristics to determine if significant differences were present between the single- and double-application groups. Descriptive statistics are presented as mean ± standard deviation or median and interquartile range [IQR] for continuous variables and counts (%) for categorical variables. Given the non-parametric nature of the data and small sample size, the Wilcoxon signed-rank test and the Mann–Whitney U test were used to analyze changes in scores from baseline to follow-up within and between each group. The Jonckeere–Terpstra (J-T) test assessed ordered trends across categorical groups. This nonparametric test was selected as an alternative to post hoc ANOVA pairwise comparisons since the data did not meet normality assumptions. Age categories (20–29, 30–39, 40–49, 50–59, 60–69, 70–79, 80–89, and 90–99). Negative differences in scores indicated greater improvement, whereas positive values reflected less improvement (except for the QOLS). This study performed no imputation, and all data sets were complete. The threshold of significance was set to *p* < 0.05, and all statistical tests were two-tailed. Significant values are bolded in the tables below. The correlation coefficient, *r*, was computed for all values to indicate effect size. Analyses were performed using SPSS Statistics (Version 31, IBM Corp, Armonk, NY, USA).

## 3. Results

### 3.1. Participants

The participant group includes 26 patients, 62% male and 38% female. Based on the severity of the defect and the physician’s discretion, 18 patients (9 males and 9 females) received a single application, and eight (7 males and 1 female) received two applications. Patient groups were divided by dosage. All patients receiving single applications reported their final scores at the 90-day visit. In the double-application group, final patient scores varied depending on when they received their second application. Data sets for the double-application group were considered complete with one additional follow-up after their final WJ application. Three out of eight patients reported their last visit at the 120-day mark, and five reported final scores at the 90-day mark. To account for the varying data sets, the percentage change and total score difference were determined using the last data set provided, irrespective of where they landed in the timeline. Baseline demographics did not differ significantly between the single and double-application groups. The mean age was 77.3 years in the single-application group and 70.7 years in the double-application group (*p* = 0.08, Kruskal–Wallis). Mean body mass index (BMI) was significantly greater in the double-application group (38.4 kg/m^2^) than in the single-application group (26.1 kg/m^2^) (*p* = 0.04, Kruskal–Wallis). Although significant differences were found between applications, only three out of eight patients reported BMI in the double-application group, which skews any conclusions based on the result. Gender distribution was 50% male in the single-application group and 88% male in the double-application group, with no statistically significant difference (*p* = 0.07, Pearson χ^2^). Several demographic factors, including age and BMI, were identified as potential confounders, which may influence application response and reported outcomes. Patient demographics are displayed in Table 1.

### 3.2. Primary Outcomes

To understand the results, one must note that a lower NPRS and WOMAC score corresponds to positive change, but a higher QOLS score reflects improvement. Of the 26 initial patients, eighteen received a single application, and eight received two sequential applications. Both groups’ mean scores demonstrated a consistent reduction in the NPRS and WOMAC scores across all follow-up visits, regardless of the number of applications. The percent improvement from initial to last reported data set for each category is presented in Table 2. The single-application cohort reported overall score improvement in 5 out of 16 patients in NPRS scores and 12 out of 18 patients for total WOMAC and QOLS scores. For patients with two applications, overall score improvement was reported in 2 out of 3 patients in NPRS scores, 4 out of 8 patients in total WOMAC scores, and 7 out of 8 patients in QOLS scores.

**Figure 1:**
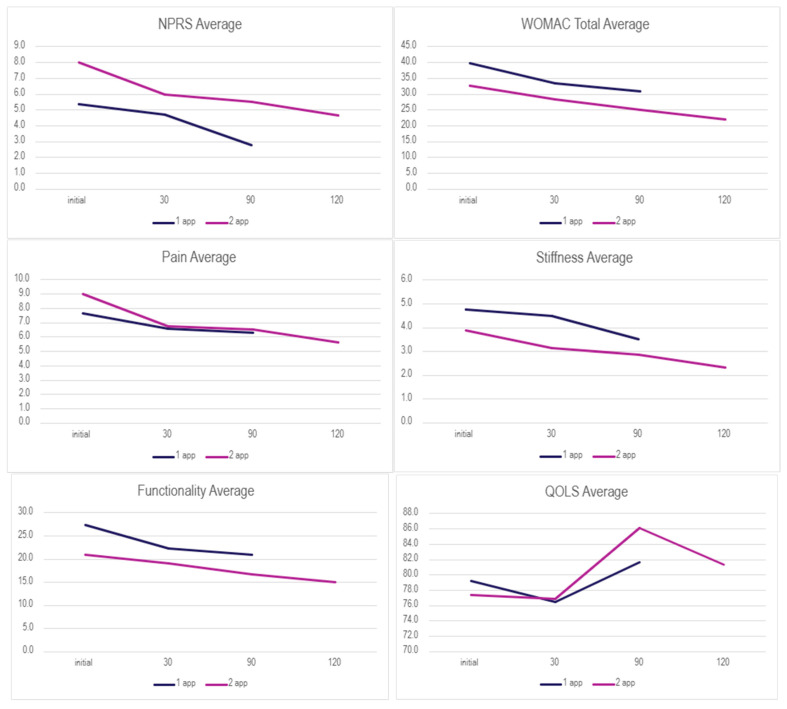
Percent improvement for each scale by number of applications from the initial to the final visit.

The Wilcoxon Signed-rank test was performed on both application groups between the initial and 30-day visits, initial and 90-day visits, and the initial and 120 visits for the double-application group. The small sample size presents limitations in the statistical strength of the test results; however, several categories displayed significant changes in scores (Table 3 and Table 4). For the single-application group (*n* = 16 for NPRS analysis), a Wilcoxon signed-rank test revealed a statistically significant reduction in NPRS scores from baseline (Md = 5, IQR: 4.25–6.75) to the 90-day visit (Md = 3, IQR: 0–5), Z = −2.032, *p* = 0.042, with a strong effect size (r = −0.51). Other scales showed improvements that did not reach statistical significance, likely due to limited sample size. In the double-application group, significant improvement was found in QOLS scores from baseline (Md = 81.5, IQR: 64.5–93.25) to the 90-day visit (Md = 86, IQR: 73–95), Z = −2.00, *p* = 0.045, r = −0.50 (*n* = 7). The very small sample size at the 120-day visit (*n* = 1–3) precluded meaningful statistical analysis for that time point. Descriptive statistics for each application group can be found in Table A1. In conjunction with the WSRT, the Mann–Whitney U test was performed to identify statistically significant values between dosage groups. Initial, final, and overall change in scores were analyzed for possible significance (Table 5). The evaluation of the mean scores between the two subgroups is visualized in Figure 1, showing common patterns of decreasing scores in both groups.

**Figure 2:**
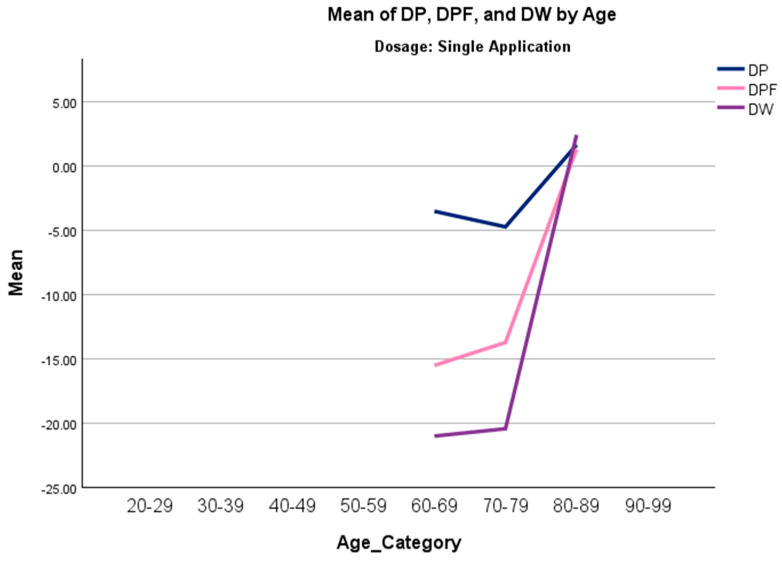
Line graph of median overall change in pain (DP), physical function (DPF), and total WOMAC (DW) scores across all age categories (20–29, 30–39, 40–49, 50–59, 60–69, 70–79, 80–89, 90–99) in the single-application group.

A Jonckheere–Terpstra (J-T) analysis was conducted to assess whether there were order trends in the overall change in scores in all measures across age categories. In the single-application group, the results indicated statistically significant trends in overall change in pain, physical function, and total WOMAC scores, suggesting that older individuals experienced less improvement (Table 6). Trends in the double-application group were not significant. Figure 2 displays a line chart for significant trends in the single-application group of the J-T test. Other covariates, such as gender and BMI, were also tested, but did not significantly predict improvement or had excessive amounts of missing data.

## 4. Discussion

The results of this research study suggest clinical benefits of applying 150 mg WJ tissue allografts to supplement tissue defects associated with posterior tibial tendon degeneration cases that were unresponsive to standard-of-care interventions. Table 2 displays the overall rate of improvement in both application categories, and Figure 1 displays the mean scores over time across each group for each scale. The Wilcoxon Signed-Rank Test (WSRT) was performed on both dosage groups to identify any statistically significant differences within each dosage group from the initial visit to the follow-ups. Notably, a statistically significant improvement in pain (NPRS) was observed in the single-application group between the initial and final follow-ups. This demonstrates that overall pain significantly dropped, indicating a positive trend of improvement in the single-application group. The double-application group revealed a significant increase in the QOLS from the initial to 90-day visits, suggesting improvement in overall quality of life despite no significance being detected for any other scale. Only three patients submitted data at the 120-day mark due to differing dates of the second application, which could explain the drop in the mean QOLS score after 90 days as well as the lack of significance between initial and final QOLS scores. The QOLSs revealed the lowest rate of improvement in both groups; this may be due to their low correlation to physical health status, although it is validated for diverse patient groups distinct from health status [20]. Although the cohort average showed improvement and reported significantly improved quality of life, confirmation of clinical success across the dosage group cannot be made. No significant differences were found between dosage groups using the Mann–Whitney U test; therefore, improvement based on dosage protocol cannot be determined.

A Jonckheere–Terpstra (J-T) analysis was conducted to investigate monotonic trends in overall change in scores across age categories. A significant positive trend was observed in three scales (DP, DPF, and DW) in the single-application group, indicating that younger patients experienced greater improvement than older patients. In contrast, age was not observed to have a significant trend in the double-application group, suggesting that multiple applications might mitigate the limited results due to biological and structural limitations that become prominent as individuals age [21]. Although this study offers important observations, generalizability is limited, and further randomized controlled trials are necessary before firm conclusions can be drawn.

These findings must be interpreted with caution due to several important limitations. First, the lack of a control group means we cannot rule out the potential effects of the natural history of the condition, regression to the mean, or the placebo effect associated with an invasive injection procedure. Second, the small sample size, particularly in the double-application subgroup (*n* = 6), greatly limits the statistical power and the generalizability of the findings. Third, the retrospective design introduces potential for selection and recall bias. Fourth, the variable follow-up rates and missing data may have influenced the results. Fifth, the lack of long-term data beyond 120 days and the absence of subgroup analyses for comorbidities or physical activity limit the understanding of WJ applications and do not establish whether the benefits are maintained over time. Future studies with comparative analysis, extended observation period, and increased patient pools are warranted to validate outcomes for the clinical potential of WJ.

Despite these limitations, the biological plausibility of WJ tissue allografts in homologous use sites is strongly supported by the analogous collagenous structure found in both the posterior tibial tendon and WJ. The collagenous framework of the posterior tibial tendon consists predominantly of collagen type I fibers, with minor compositions of collagen III and V [15]. These matrices are arranged in organized bundles with crosslinking collagen fibers parallel to the tendon axis, and failure to maintain these structures allows the thickening of the tendon, stiffness, pain, instability, and the increased risk of tendinopathy [22,23]. Similarly, WJ consists of rich concentrations of collagen types I, II, III, and V, with other beneficial extracellular matrix components [13,14]. The tissue matrix of WJ offers protection from tensile stress and cushioning of the umbilical cord, mirroring critical functions of tendons in maintaining joint movement and withstanding mechanical stress [13,23]. WJ tissue allografts can successfully be used to supplement cases of posterior tibial tendon degeneration, allowing for healthy collagen structures to interlock with the native matrices.

The exploratory results provide preliminary evidence of novel regenerative medicine protocols for practicing physicians seeking alternative conservative care options to avoid postoperative risks and complications. According to current literature and the physicians who conducted this study, standard surgical procedures for individuals with posterior tibial tendon degeneration have not been reported as preferred methods of managing posterior tibial tendon degeneration [24,25]. Postoperative complications often include thromboembolic events, infection, wound dehiscence, and neurologic injury [11]. Platelet-rich plasma (PRP) therapy, an autologous regenerative intervention, has garnered substantial clinical interest; however, recent studies underscore its inconsistent effectiveness and the lack of standardized treatment protocols [24]. The use of PRP in tendon degeneration has resulted in negative outcomes and adverse events in the current literature [26,27,28]. Prolotherapy, an emerging method focusing on vascular growth and tissue repair by an injection of an irritant solution into the injured tissue, has shown benefits in lower extremity sites; however, these appear to be limited to short-term outcomes, and recent studies reported no significant pain improvements compared with other conservative therapies [29,30,31]. The integration of WJ allografts into regenerative medicine has been demonstrated in other use sites such as rotator cuff tears, hip and knee osteoarthritis, and sacroiliac cartilaginous defects [17,18,19,32]. Results from each study have demonstrated increases in functionality with decreases in pain and stiffness with no adverse events.

The structural and functional parallels between WJ and the posterior tibial tendon and positive trends in patient improvement discussed in this observational study demonstrate that the application of WJ tissue allografts to homologous use sites is a promising alternative to current conventional care. WJ may help circumvent the need for operative management by facilitating earlier intervention and promoting the preservation of native tendon morphology. The promising safety profile and positive trend in outcomes provide a rationale for future research. Future studies prioritizing prospective, randomized controlled trials comparing WJ to placebo or active comparators like PRP, with larger sample sizes, standardized dosing protocols, and extended follow-up periods, will be instrumental in validating the clinical potential of WJ in its application to refractory posterior tibial tendon degeneration.

## 5. Conclusions

This pilot observational study of 26 patients with PTTD found that percutaneous application of Wharton’s jelly allografts was associated with improvements in pain, stiffness, physical function, and quality of life over a 90–120-day period, with no reported adverse events. The significant improvement in pain scores is encouraging. However, the retrospective nature, small sample size, and absence of a control group prevent definitive conclusions from being drawn regarding efficacy. Despite the limitations, these preliminary findings support the safety and feasibility of WJ allografts for PTTD and highlight the imperative for rigorous, randomized controlled trials to validate these results and define the role of this regenerative therapy in the clinical management of PTTD.

## Figures and Tables

**Table 1:** Baseline characteristics of the patients involved in the study.

Variable	1 App (*n* = 18)	2 App (*n* = 8)	Total (*n* = 26)	*p*-Value
**Age [years]**				
50–59	0	0	0	
60–69	2 (11%)	3 (38%)	5 (19%)	
70–79	7 (39%)	4 (50%)	11 (42%)	
80–89	9 (50%)	1 (12%)	10 (39%)	
Mean Age	77.3	70.7	75.0	0.080 ^1^
**BMI [kg/m^2^]**				
Healthy (18.5–24.9)	4 (22%)	0	4 (15%)	
Overweight (25.0–29.9)	3 (17%)	0	3 (12%)	
Obese (>30)	1 (6%)	3 (38%)	4 (15%)	
Missing	10 (56%)	5 (62%)	15 (58%)	
Mean BMI	26.1	38.4	30.0	0.040 ^2^
**Gender**				0.070 ^3^
Male	9 (50%)	7 (88%)	16 (62%)	
Female	9 (50%)	1 (12%)	10 (38%)	

^1^ Kruskal–Wallis test. ^2^ Kruskal–Wallis test (BMI continuous). ^3^ Chi-square test.

**Table 2:** Percent improvement in each scale by the number of applications from the initial to the last reported data set.

Scale	1 App	2 App
NPRS	48.32%	40.74%
WOMAC Total	22.73%	5.50%
Pain	18.12%	21.88%
Stiffness	26.74%	27.08%
Functionality	23.33%	6.67%
QOLS	3.02%	2.60%

**Table 3:** Test statistics for single-application group.

Single-Application Test Statistics ^a^						
	NPRS2–NPRS1	NPRS3–NPRS1	P2–P1	P3–P1	S2–S1	S3–S1
Z	−1.078 ^b^	−2.032 ^b^	−0.739 ^b^	−1.425 ^b^	−0.876 ^b^	−1.511 ^b^
Asymp. Sig. (2-tailed)	0.281	**0.042**	0.460	0.154	0.381	0.131
r	−0.22	−0.51	−0.12	−0.24	−0.15	−0.25
	PF2–PF1	PF3–PF1	W2–W1	W3–W1	Q2–Q1	Q3–Q1
Z	−1.706 ^b^	−1.888 ^b^	−1.421 ^b^	−1.729 ^b^	−0.796 ^b^	−1.135 ^c^
Asymp. Sig. (2-tailed)	0.088	0.059	0.155	0.084	0.426	0.256
r	−0.28	−0.31	−0.24	−0.29	−0.13	−0.19

Key: NPRS: Numerical Pain Rating Scale; P: WOMAC—Pain; S: WOMAC—Stiffness; PF: WOMAC—Physical Function; W: WOMAC—Total; Q: Quality of life scale; 1: Initial Visit; 2: 30-Day Visit; 3: 90-Day Visit. ^a^ Wilcoxon Signed Ranks Test. ^b^ Based on positive ranks. ^c^ Based on negative ranks. Significant values are in bold.

**Table 4:** Test statistics for double-application group.

Double-Application Test Statistics ^a^							
	NPRS2–NPRS1	NPRS3–NPRS1	P2–P1	P3–P1	P4–P1	S2–S1	
Z	−1.000 ^b^	−1.342 ^b^	−1.581 ^b^	−1.529 ^b^	−1.604 ^b^	−0.962 ^b^	
Asymp. Sig. (2−tailed)	0.317	0.180	0.114	0.126	0.109	0.336	
r	−0.50	−0.67	−0.40	−0.38	−0.65	−0.24	
	S3–S1	S4–S1	PF2–PF1	PF3–PF1	PF4–PF1	W2–W1	W3–W1
Z	−1.372 ^b^	−1.604 ^b^	−0.140 ^c^	−0.734 ^b^	−1.069 ^b^	−0.420 ^b^	−1.363 ^b^
Asymp. Sig. (2−tailed)	0.170	0.109	0.889	0.463	0.285	0.674	0.173
r	−0.34	−0.65	−0.04	−0.18	−0.45	−0.11	−0.34
	W4–W1	Q2–Q1	Q3–Q1	Q4–Q1			
Z	−1.069 ^b^	0.000 ^d^	−2.003 ^c^	−1.604 ^c^			
Asymp. Sig. (2−tailed)	0.285	1.000	**0.045**	0.109			
r	−0.44	0.00	−0.50	−0.65			

Key: NPRS: Numerical Pain Rating Scale; P: WOMAC—Pain; S: WOMAC—Stiffness; PF: WOMAC—Physical Function; W: WOMAC—Total; Q: Quality of life scale; 1: Initial Visit; 2: 30-Day Visit; 3: 90-Day Visit; 4: 120-Day Visit. ^a^ Wilcoxon Signed Ranks Test. ^b^ Based on positive ranks. ^c^ Based on negative ranks. ^d^ The sum of negative ranks equals the sum of positive ranks. Significant values are in bold.

**Table 5:** Test statistics for single- vs. double-application groups.

Single vs. Double Applications							
	NPRS1	P1	S1	PF1	W1	Q1	FNPRS
Mann–Whitney U	23.000	68.000	54.500	55.000	60.500	68.500	13.500
Wilcoxon W	29.000	239.000	90.500	91.000	96.500	104.500	19.500
Z	−0.114	−0.223	−0.982	−0.946	−0.640	−0.195	0.000
Asymp. Sig. (2-tailed)	0.909	0.823	0.326	0.344	0.522	0.846	1.000
Exact Sig. [2*(1-tailed Sig.)]	0.958 ^b^	0.849 ^b^	0.338 ^b^	0.367 ^b^	0.531 ^b^	0.849 ^b^	1.000 ^b^
	FP	FS	FPF	FW	FQ	DNPRS	DP
Mann–Whitney U	64.500	57.500	58.500	58.500	65.500	5.500	64.500
Wilcoxon W	100.500	93.500	94.500	94.500	236.500	8.500	100.500
Z	−0.419	−0.814	−0.752	−0.751	−0.362	−0.848	−0.419
Asymp. Sig. (2-tailed)	0.675	0.416	0.452	0.452	0.718	0.396	0.675
Exact Sig. [2*(1-tailed Sig.)]	0.683 ^b^	0.429 ^b^	0.461 ^b^	0.461 ^b^	0.724 ^b^	0.436 ^b^	0.683 ^b^
	DS	DPF	DW	DQ			
Mann–Whitney U	71.500	57.000	61.000	52.000			
Wilcoxon W	242.500	228.000	232.000	223.000			
Z	−0.028	−0.834	−0.612	−1.114			
Asymp. Sig. (2-tailed)	0.978	0.404	0.541	0.265			
Exact Sig. [2*(1-tailed Sig.)]	0.978 ^b^	0.429 ^b^	0.567 ^b^	0.285 ^b^			

Key: NPRS: Numerical Pain Rating Scale; P: WOMAC—Pain; S: WOMAC—Stiffness; PF: WOMAC—Physical Function; W: WOMAC—Total; Q: Quality of life scale; 1: Initial Visit; F: Last Visit; D: Difference (Final—Initial). * Grouping Variable: Dosage. ^b^ Not corrected for times.

**Table 6:** Jonckheere–Terpstra test results examining ordered trends in overall score changes for all measures across age groups.

Jonckheere–Terpstra Test ^a^							
Dosage		DNPRS	DP	DS	DPF	DW	DQ
Single Application	Number of Levels in Age_Category	3	3	3	3	3	3
	N	9	18	18	18	18	18
	Observed J-T Statistic	8.500	78.000	64.000	80.000	75.500	25.000
	Mean J-T Statistic	12.000	47.500	47.500	47.500	47.500	47.500
	Std. Deviation of J-T Statistic	4.072	11.745	11.738	11.822	11.816	11.758
	Std. J-T Statistic	−0.860	2.597	1.406	2.749	2.370	−1.914
	Asymp. Sig. (2-tailed)	0.390	**0.009**	0.160	**0.006**	**0.018**	0.056
Double Application	Number of Levels in Age_Category	2	3	3	3	3	3
	N	2	8	8	8	8	8
	Observed J-T Statistic	0.000	9.500	9.500	4.000	8.000	12.000
	Mean J-T Statistic	0.500	9.500	9.500	9.500	9.500	9.500
	Std. Deviation of J-T Statistic	0.500	3.617	3.524	3.640	3.640	3.524
	Std. J-T Statistic	−1.000	0.000	0.000	−1.511	−0.412	0.709
	Asymp. Sig. (2-tailed)	0.317	1.000	1.000	0.131	0.680	0.478

^a^ Grouping Variable: Age_Category. Significant values are in bold.

## Data Availability

The datasets presented in this article are not readily available because the data are part of an ongoing. Requests to access the datasets should be directed to Tyler Barrett.

## Appendix A

**Table A1:** Descriptive statistics for each dosage group.

Statistics							
Dosage			NPRS1	P1	S1	PF1	W1
Single Application	N	Valid	16	18	18	18	18
		Missing	2	0	0	0	0
	Mean		5.3750	7.6667	4.7778	27.3889	39.8333
	Median		5.0000	6.5000	4.5000	23.0000	32.0000
	Std. Deviation		1.85742	4.52444	2.15722	16.77933	22.19499
	Skewness		0.082	0.151	0.006	0.759	0.730
	Std. Error of Skewness		0.564	0.536	0.536	0.536	0.536
	Kurtosis		−0.096	−0.945	−1.254	−0.440	−0.616
	Std. Error of Kurtosis		1.091	1.038	1.038	1.038	1.038
	Percentiles	25	4.2500	5.2500	3.0000	15.0000	26.2500
		50	5.0000	6.5000	4.5000	23.0000	32.0000
		75	6.7500	12.2500	7.0000	40.0000	59.2500
Dosage			NPRS1	P1	S1	PF1	W1
Double Application	N	Valid	3	8	8	8	8
		Missing	5	0	0	0	0
	Mean		5.3333	8.0000	3.8750	20.8750	32.7500
	Median		5.0000	8.0000	5.0000	17.5000	31.0000
	Std. Deviation		1.52753	3.85450	2.23207	12.36860	16.50757
	Skewness		0.935	0.319	−0.821	1.290	1.113
	Std. Error of Skewness		1.225	0.752	0.752	0.752	0.752
	Kurtosis			−1.272	−0.815	1.546	1.690
	Std. Error of Kurtosis			1.481	1.481	1.481	1.481
	Percentiles	25	4.0000	4.0000	2.0000	11.5000	20.0000
		50	5.0000	8.0000	5.0000	17.5000	31.0000
		75		11.5000	5.7500	27.7500	40.7500
Statistics							
Dosage			Q1	NPRS2	P2	S2	PF2
Single Application	N	Valid	18	13	18	18	18
		Missing	0	5	0	0	0
	Mean		79.2222	4.6923	6.6111	4.5000	22.2778
	Median		82.0000	5.0000	6.0000	4.5000	19.5000
	Std. Deviation		18.78534	1.65250	4.81589	2.03643	14.07879
	Skewness		−0.191	−0.074	0.429	0.071	0.733
	Std. Error of Skewness		0.536	0.616	0.536	0.536	0.536
	Kurtosis		−0.511	−0.394	−0.931	−1.048	0.646
	Std. Error of Kurtosis		1.038	1.191	1.038	1.038	1.038
	Percentiles	25	64.5000	4.0000	3.0000	3.0000	9.7500
		50	82.0000	5.0000	6.0000	4.5000	19.5000
		75	89.2500	6.0000	10.2500	6.2500	31.2500
Dosage			Q1	NPRS2	P2	S2	PF2
Double Application	N	Valid	8	4	8	8	8
		Missing	0	4	0	0	0
	Mean		77.3750	4.7500	6.0000	3.1250	19.1250
	Median		81.5000	5.0000	6.0000	4.0000	18.5000
	Std. Deviation		18.56985	2.06155	2.07020	2.23207	8.80645
	Skewness		−0.758	−0.713	−0.902	−0.515	0.287
	Std. Error of Skewness		0.752	1.014	0.752	0.752	0.752
	Kurtosis		0.378	1.785	0.812	−1.105	1.205
	Std. Error of Kurtosis		1.481	2.619	1.481	1.481	1.481
	Percentiles	25	64.5000	2.7500	5.0000	0.5000	13.5000
		50	81.5000	5.0000	6.0000	4.0000	18.5000
		75	93.2500	6.5000	8.0000	4.7500	24.0000
Statistics							
Dosage			W2	Q2	NPRS3	P3	S3
Single Application	N	Valid	18	18	9	18	18
		Missing	0	0	9	0	0
	Mean		33.3889	76.5000	2.7778	6.2778	3.5000
	Median		29.0000	85.0000	3.0000	5.0000	3.5000
	Std. Deviation		19.30297	20.77116	2.22361	4.62481	2.33263
	Skewness		0.758	−0.076	−0.432	0.816	0.282
	Std. Error of Skewness		0.536	0.536	0.717	0.536	0.536
	Kurtosis		0.074	−0.874	−1.788	−0.014	−0.744
	Std. Error of Kurtosis		1.038	1.038	1.400	1.038	1.038
	Percentiles	25	14.0000	55.5000	0.0000	3.0000	2.0000
		50	29.0000	85.0000	3.0000	5.0000	3.5000
		75	51.2500	88.0000	5.0000	9.2500	5.2500
Dosage			W2	Q2	NPRS3	P3	S3
Double Application	N	Valid	8	8	3	8	8
		Missing	0	0	5	0	0
	Mean		28.2500	76.8750	2.6667	5.5000	2.8750
	Median		27.0000	70.0000	2.0000	5.5000	3.5000
	Std. Deviation		12.42980	13.75747	2.08167	3.02372	2.29518
	Skewness		−0.023	0.638	1.293	0.145	−0.176
	Std. Error of Skewness		0.752	0.752	1.225	0.752	0.752
	Kurtosis		0.897	−1.447		−1.506	−1.589
	Std. Error of Kurtosis		1.481	1.481		1.481	1.481
	Percentiles	25	21.2500	67.5000	1.0000	2.2500	0.2500
		50	27.0000	70.0000	2.0000	5.5000	3.5000
		75	36.7500	92.7500		8.0000	4.7500
Statistics							
Dosage			PF3	W3	Q3		
Single Application	N	Valid	18	18	18		
		Missing	0	0	0		
	Mean		21.0000	30.7778	81.6111		
	Median		19.0000	26.0000	80.5000		
	Std. Deviation		14.21681	20.42074	17.38200		
	Skewness		0.222	0.244	−0.017		
	Std. Error of Skewness		0.536	0.536	0.536		
	Kurtosis		−0.999	−1.055	−0.034		
	Std. Error of Kurtosis		1.038	1.038	1.038		
	Percentiles	25	10.7500	16.0000	73.5000		
		50	19.0000	26.0000	80.5000		
		75	33.2500	51.0000	91.0000		
Dosage			PF3	W3	Q3	NPRS4	P4
Double Application	N	Valid	8	8	7	1	3
		Missing	0	0	1	7	5
	Mean		16.6250	25.0000	86.1429	1.0000	4.6667
	Median		17.5000	25.0000	86.0000	1.0000	5.0000
	Std. Deviation		9.21082	14.02039	12.38855		2.51661
	Skewness		−0.040	0.070	−0.077		−0.586
	Std. Error of Skewness		0.752	0.752	0.794		1.225
	Kurtosis		−0.618	−0.727	−0.916		
	Std. Error of Kurtosis		1.481	1.481	1.587		
	Percentiles	25	7.0000	10.0000	73.0000	1.0000	2.0000
		50	17.5000	25.0000	86.0000	1.0000	5.0000
		75	23.2500	36.0000	95.0000	1.0000	
Statistics							
Dosage			S4	PF4	W4	Q4	
Double Application	N	Valid	3	3	3	3	
		Missing	5	5	5	5	
	Mean		2.3333	15.0000	22.0000	81.3333	
	Median		3.0000	10.0000	18.0000	86.0000	
	Std. Deviation		2.08167	12.28821	16.37071	8.96289	
	Skewness		−1.293	1.528	1.034	−1.708	
	Std. Error of Skewness		1.225	1.225	1.225	1.225	
	Kurtosis						
	Std. Error of Kurtosis						
	Percentiles	25	0.0000	6.0000	8.0000	71.0000	
		50	3.0000	10.0000	18.0000	86.0000	
		75					
